# Bruno 1 remodels P-bodies to protect select mRNAs during the early ER stress response

**DOI:** 10.26508/lsa.202603806

**Published:** 2026-08-04

**Authors:** Samantha N Milano, Livia V Bayer, Julie J Ko, Gwendolyn S Posner, Albina H Granovsky, Diana P Bratu

**Affiliations:** 1 Department of Biological Sciences, Hunter College, City University of New York , NY, USA; 2 Program in Molecular, Cellular, and Developmental Biology, The Graduate Center, City University of New York , NY, USA

## Abstract

P-bodies are cytoplasmic membraneless organelles involved in mRNA storage, yet their role in cellular stress responses remains unresolved. Here, we demonstrate that P-bodies are remodeled during the early response to ER stress throughout *Drosophila melanogaster* oogenesis. Notably, this remodeling occurs within minutes of stress induction and precedes stress granule formation. This early remodeling is characterized by changes in P-body morphology and internal organization and promotes selective mRNA storage. Mechanistically, we find that this process is driven by transcriptional up-regulation of the RNA-binding protein, Bruno 1, downstream of ATF4-dependent stress signaling, thereby establishing a connection between the unfolded protein response and condensate regulation. Consistent with this model, loss of Bruno 1 abolishes, whereas its overexpression enhances, P-body remodeling, demonstrating that stress-induced changes in RNA-binding protein levels can reprogram condensate properties. Together, our findings reveal that P-bodies function as stress-responsive hubs enabling selective preservation of essential mRNAs during ER stress. More broadly, this work uncovers a previously unrecognized mechanism by which stress signaling pathways reorganize cytoplasmic architecture to shape mRNA fate.

## Abstract (precis)

During *Drosophila melanogaster* oogenesis, activation of the unfolded protein response induces ATF4-dependent Bruno 1 expression, reshaping P-body architecture and composition to safeguard specific mRNAs from stress-induced degradation before stress granule formation.

## Introduction

Cells rely on tightly coordinated mechanisms to modulate mRNA fate during cellular stress, such as heat shock, nutrient deprivation, or hypoxia (Fulda et al, 2010). To survive these perturbations, cells must rapidly adjust mRNA stability, localization, and translation (Yamasaki & Anderson, 2008; Srikantan & Gorospe, 2010). In many cases, translationally stalled mRNAs are sequestered within well-characterized cytoplasmic membraneless organelles (MLOs) called stress granules, where they are preserved until conditions improve and translation can resume (Protter & Parker, 2016). Another class of cytoplasmic MLOs, processing bodies (P-bodies), is enriched in translationally repressed mRNAs and RNA-binding proteins that function in mRNA storage, silencing, and turnover (Luo et al, 2018). In contrast to stress granules, P-bodies are present under basal conditions. Although the role of stress granules in cellular stress adaptation has been extensively investigated, the contribution of P-bodies to stress responses, particularly during the early stages of ER stress, remains poorly understood.

Accumulation of unfolded or misfolded proteins within the ER lumen causes ER stress and activates the unfolded protein response (UPR) (Read & Schröder, 2021). The UPR engages three primary signaling branches mediated by X-box–binding protein 1 (XBP1), and activating transcription factors 4 and 6 (ATF4 and ATF6), which together act as transcription factors and reprogram gene expression to increase ER chaperone production, reduce ER load with general mRNA degradation, suppress global protein synthesis, and restore ER proteostasis (Cao & Kaufman, 2012; Hetz et al, 2020). The ER stress response is generally cytoprotective; however, prolonged or unresolved ER stress can become detrimental, leading to autophagy or chronic pathological states (Yorimitsu & Klionsky, 2007; Yoshida, 2007). As such, persistent ER stress is implicated in the etiology of numerous diseases, including Alzheimer’s disease, Parkinson’s disease, cancer, and diabetes (Cnop et al, 2012; Yadav et al, 2014; Mou et al, 2020; Ajoolabady et al, 2022). Understanding how cells coordinate the ER stress response at the level of mRNA regulation is therefore of fundamental biological and clinical importance.

Prior work from our group and others indicates a functional connection between the ER and P-bodies (Kilchert et al, 2010; Lee et al, 2020; Milano et al, 2024). P-bodies are conserved across species and cell types and are constitutively present in the cytoplasm, positioning them to act as early responders to ER stress (Luo et al, 2018). These MLOs primarily house translationally repressed transcripts and function as organizational hubs in which multiple mRNA species can be recruited, stored, and released in a regulated manner (Standart & Weil, 2018). P-bodies are highly dynamic structures that can alter their mRNA content in response to cellular cues, thereby promoting selective transcript storage or release depending on the cellular environment (Sankaranarayanan et al, 2021).

P-bodies form via liquid–liquid phase separation, which occurs when local concentration of mRNAs and associated proteins becomes sufficiently enriched to favor the formation of a distinct condensed phase (Brangwynne et al, 2009; Elbaum-Garfinkle et al, 2015; Banani et al, 2017). The molecular composition of these condensates influences their emergent material properties, which are governed by networks of weak, multivalent interactions, including protein:protein, RNA:RNA, and RNA:protein interactions (Mittag & Parker, 2018; Borcherds et al, 2021; Wadsworth et al, 2024).

P-bodies exist along a continuum of material states ranging from liquid-like to more solid-like. In vitro, nascent condensates often exhibit liquid-like properties but can progressively mature into more rigid assemblies over time as intracondensate interactions stabilize (Jawerth et al, 2020). In vivo, cells appear to actively regulate these material properties, preventing aberrant solidification and maintaining functional condensate dynamics (Kroschwald et al, 2015; Lin et al, 2015; Jain et al, 2016). Emerging evidence suggests that the P-body material state influences function: more solid-like P-bodies may preferentially support long-term transcript storage, whereas more liquid-like condensates may facilitate rapid transcript exchange, recruitment, and release for translation or degradation (Sankaranarayanan et al, 2021; Milano et al, 2026).

The material properties of P-bodies are partially determined by the composition of their constituent proteins. Many P-body proteins contain intrinsically disordered regions (IDRs), which promote liquid–liquid phase separation (Schütz et al, 2017; Protter et al, 2018). In *Drosophila melanogaster*, several conserved P-body–associated proteins have been identified, including: Me31B (DDX6), Trailer Hitch (Tral) (Lsm14 homolog), Cup (eIF4E-binding protein), and Bruno 1 (a member of the CELF family). Me31B is a DEAD-box RNA helicase that represses translation and exhibits high interaction valency, enabling multivalent protein:protein and protein:RNA interactions (de Valoir et al, 1991; Nakamura et al, 2001). Tral is an RNA-binding protein–enriched IDRs, which promote phase separation and modulate the emergent material properties of biomolecular condensates (Tritschler et al, 2008; Sankaranarayanan et al, 2021). Cup is a large IDR-containing protein that binds eIF4E and competitively inhibits assembly of the translation initiation complex, thereby preventing ribosome recruitment. Cup is recruited to target mRNAs independently, as well as via Bruno 1, which recognizes Bruno response elements within transcripts (Kim-Ha et al, 1995; Piccioni et al, 2005; Bayer et al, 2023). Collectively, these proteins, together with associated mRNAs, drive the formation of phase-separated P-bodies that function as selective hubs for post-transcriptional gene regulation.

Here, we investigate the previously undefined role of P-bodies in the cellular response to ER stress. The contribution of post-transcriptional regulation to maintaining cellular homeostasis during the earliest phases of ER stress remains poorly understood. Thus, studying the early stages of stress response, when timely changes in mRNA fate can shape adaptive or pathological outcomes, is of value. Interestingly, we find that P-bodies respond rapidly to ER stress, before stress granule formation, by undergoing changes in material state and selectively accumulating translationally repressed mRNAs. This response promotes the stabilization of specific mRNAs and depends on signaling through the ATF4 branch of the UPR and up-regulation of Bruno 1. Together, our findings identify P-bodies as early regulators of mRNA fate during ER stress and reveal a previously unappreciated layer of post-transcriptional control in the UPR.

## Results

### P-body morphology rapidly changes upon ER stress induction

**Figure 1.**
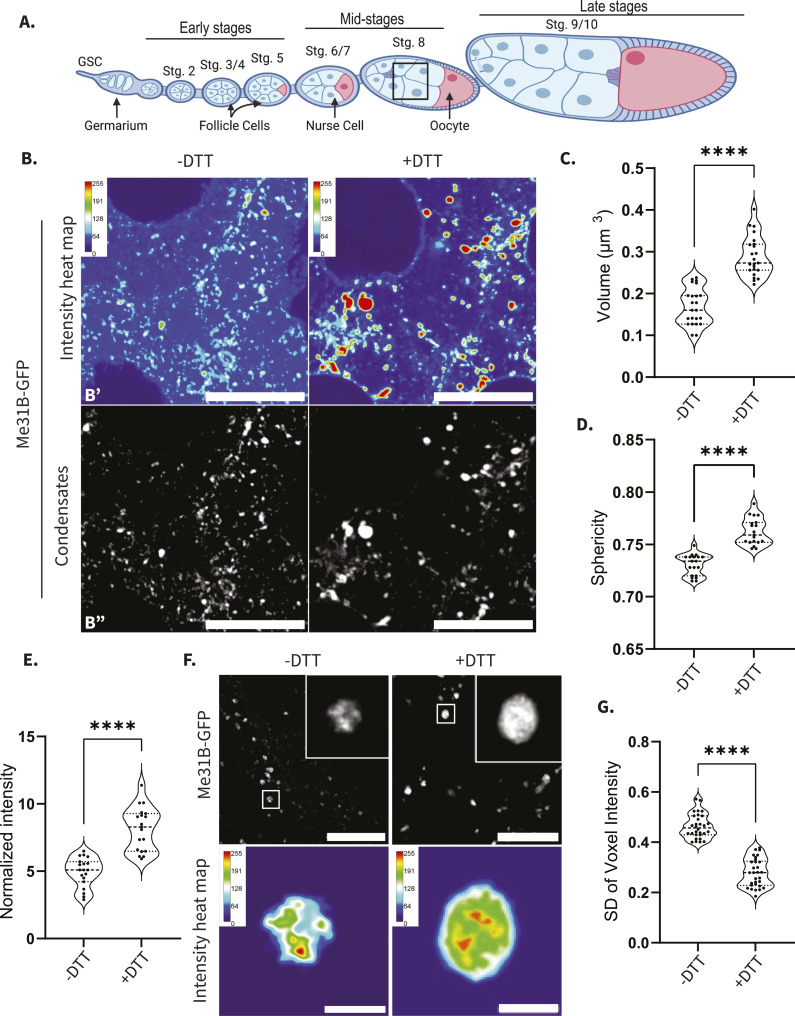
P-body morphology rapidly changes upon ER stress induction. **(A)** Schematic of a fly ovariole. Developmental stages progress from left to right. The oocyte (pink) and the nurse cells (light blue) are surrounded by somatic follicles (darker blue). The outlined region of interest (black square) indicates the nurse cell region visualized for all zoomed image panels in this study. **(B)** Endogenously expressed Me31B-GFP visualized (B’) with an intensity heat map and (B”) with the cytoplasmic signal removed. Images are XY projections of 5 optical Z-slices of 0.3 µm. Scale bars are 20 µm. **(C)** Volume quantifications comparing Me31B-GFP condensates in −DTT- and +DTT-incubated egg chambers (n = 23). **(D)** Sphericity quantifications comparing Me31B-GFP condensates in −DTT- and +DTT-incubated egg chambers (n = 19). **(E)** Voxel intensity quantifications comparing Me31B-GFP condensates in −DTT- and +DTT-incubated egg chambers (n = 19). **(F)** Visualization of endogenous Me31B-GFP via STED. Images are XY projections of 5 optical Z-slices of 0.22 µm. The scale bar is 5 µm (1 µm for the zoomed inset). **(G)** SD of voxel intensity values for Me31B-GFP condensates in −DTT- and +DTT-incubated egg chambers (n = 30). For all plots, each data point represents the average value of all P-bodies detected in an image. Significance was assessed using Mann–Whitney statistical tests. Error bars represent the SD. ****P < .0001.

To investigate how ER stress influences P-body organization, we used *D. melanogaster*, a well-established system for dissecting ER stress responses because of the high conservation of key regulatory pathways (Ryoo & Steller, 2007; Ryoo, 2015). We focused on egg chambers, as the female germline has long served as a powerful model for studying RNA regulation and biomolecular condensates. Each egg chamber comprises 16 interconnected germline cells encapsulated by a layer of somatic follicle cells. Among these germline cells, one differentiates into a transcriptionally silent oocyte, whereas the remaining 15 become transcriptionally active nurse cells that synthesize and supply all maternal mRNAs required for oocyte development (Fig 1A) (McLaughlin & Bratu, 2015). The high transcriptional output of nurse cells, coupled with the need for extensive post-transcriptional regulation and long-range mRNA transport to the oocyte, makes this system particularly well suited for studying P-body dynamics. This prodigious biosynthetic demand requires rapid cellular growth and sustained protein production, rendering nurse cells particularly vulnerable to disruptions in proteostasis and cellular stress. Development initiates in the germarium and proceeds through 14 morphologically defined stages before embryogenesis. These stages are broadly categorized as early (stages 1–5), mid (stages 6–8), and late (stages 9–14) oogenesis. For consistency, most of our quantitative analyses were performed in nurse cells from mid-stage egg chambers (Fig 1A, black box).

To induce ER stress, ovaries were dissected and incubated ex vivo in Schneider’s media supplemented with 5 mM dithiothreitol (DTT), a well-established ER stressor that disrupts disulfide bond formation and activates the UPR (Schröder, 2008). P-bodies were visualized with endogenously tagged Me31B-GFP. Although Me31B is highly enriched within P-bodies, it is also distributed throughout the cytoplasm; notably, studies of its mammalian homolog have shown that more than 80% of the protein resides outside of condensates (Ernoult-Lange et al, 2012). To enhance visualization of the P-body–associated signal, we removed the diffuse cytoplasmic Me31B signal (voxel intensities below 70) and displayed only condensate-localized Me31B throughout the study (Fig 1B).

After 30 min of DTT treatment (+DTT), Me31B-marked condensates exhibited an ∼71% increase in volume (from 0.169 to 0.289 μm^3^) and an ∼4.36% increase in sphericity (from 0.730 to 0.762) relative to control egg chambers incubated in Schneider’s media alone (−DTT) (Fig 1C and D). Even modest increases in sphericity have been linked to reduced intracondensate bonds and are consistent with remodeling of condensate architecture (Sankaranarayanan et al, 2021; Bayer et al, 2023).

**Figure S1.**
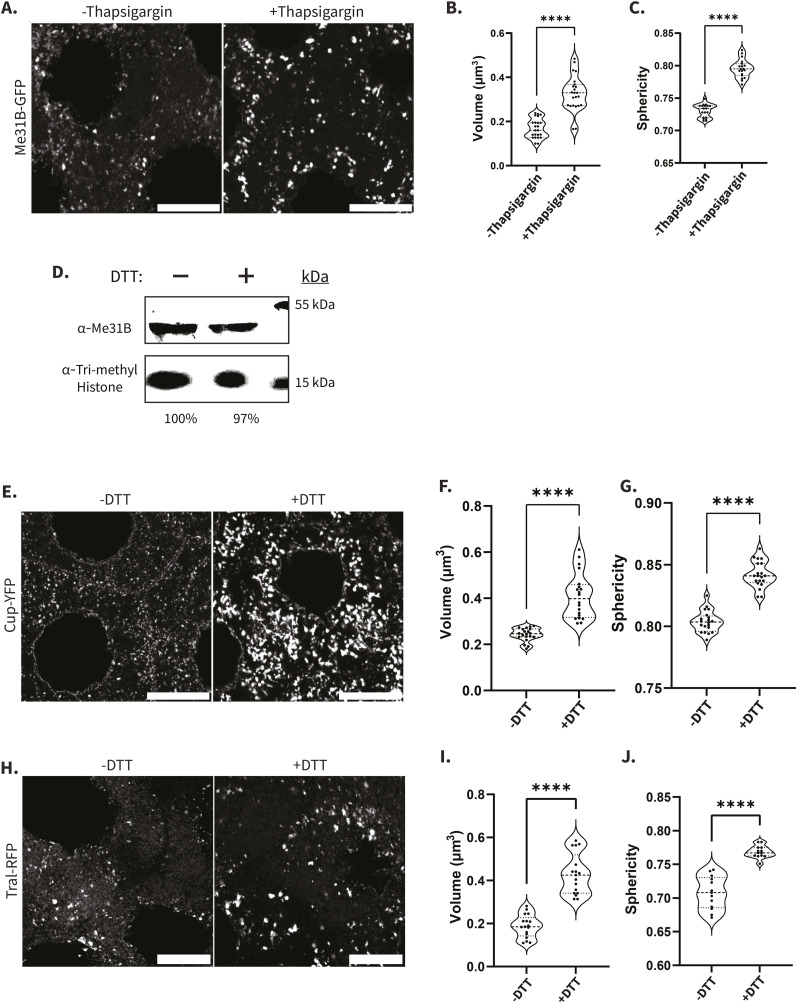
P-body morphology rapidly changes upon ER stress induction. **(A)** Visualization of endogenously expressed Me31B-GFP in egg chambers incubated ex vivo in Schneider’s media alone (−thapsigargin) or in Schneider’s media supplemented with thapsigargin (+thapsigargin). Images are XY projections of 5 optical Z-slices of 0.3 µm. Scale bars are 20 µm. **(B)** Volume quantifications comparing Me31B-GFP condensates in -thapsigargin- and +thapsigargin-incubated egg chambers (n = 23). **(C)** Sphericity quantifications comparing Me31B-GFP condensates in −thapsigargin- and +thapsigargin-incubated egg chambers (n = 19). **(D)** Western blot analysis of Me31B in −DTT- and +DTT-incubated egg chambers (Tri-methyl-Histone—loading control). **(E)** Visualization of endogenous Cup-YFP condensates in −DTT- and +DTT-incubated egg chambers. Images are XY projections of 5 optical Z-slices of 0.3 µm. Scale bars are 20 µm. **(F)** Volume quantifications comparing Cup-YFP condensates in −DTT- and +DTT-incubated egg chambers (n = 20). **(G)** Sphericity quantifications comparing Cup-YFP condensates in −DTT- and +DTT-incubated egg chambers (n = 20). **(H)** Visualization of endogenous Tral-RFP condensates in −DTT- and +DTT-incubated egg chambers. Images are XY projections of 5 optical Z-slices of 0.3 µm. Scale bars are 20 µm. **(I)** Volume quantifications comparing Tral-RFP condensates in egg chambers incubated for 30 min in −DTT and +DTT (n = 20). **(J)** Sphericity quantifications comparing Tral-RFP condensates in egg chambers incubated for 30 min in −DTT and +DTT (n = 20). For all plots, each data point represents the average value of all P-bodies detected in an image. Significance was assessed using Mann–Whitney statistical tests. Error bars represent the SD. *P < .1, ****P < .0001.

To confirm that these effects were due to ER stress rather than a DTT-specific artifact, we induced ER stress using an independent mechanism. Treatment with 1 μM thapsigargin, which perturbs ER calcium homeostasis, similarly resulted in an ∼94% increase in P-body volume (from 0.168 to 0.326 µm^3^) and an ∼8.90% increase in sphericity (from 0.730 to 0.795) after 30 min (Fig S1A–C; Inesi & Sagara, 1992).

We next asked whether the observed increase in P-body size reflected elevated Me31B protein levels or a redistribution of the existing protein. Western blot analysis revealed that total Me31B protein levels were unchanged after ER stress induction, indicating that the morphological changes occur independently of altered Me31B expression levels (Fig S1D). Consistent with the observed increase in condensate size, normalized Me31B-GFP intensity within P-bodies also increased by ∼62.29% after ER stress, further suggesting enhanced partitioning of Me31B into condensates rather than increased protein abundance (Fig 1E).

To further confirm that these effects reflected general P-body remodeling rather than protein-specific behavior, we examined additional P-body components, Cup and Tral. Endogenously expressed Cup-YFP–labeled condensates increased in volume by ∼68.85% and in sphericity by ∼4.74% upon ER stress induction (Fig S1E–G). Similarly, endogenously expressed Tral-RFP condensates increased in volume by ∼128.09% and in sphericity by ∼8.59%, supporting the conclusion that ER stress broadly alters P-body morphology (Fig S1H–J).

To investigate whether these morphological changes were accompanied by alterations in internal P-body organization, we employed STED super-resolution microscopy to resolve intracondensate structure (Fig 1F). ER stress induction led to a marked change in the spatial distribution of Me31B-GFP fluorescent signal within individual P-bodies. To quantify this, we measured the standard deviation (SD) of voxel intensities within each condensate. Highly structured condensates exhibit heterogeneous fluorescence intensities and therefore higher voxel intensity SDs, whereas less internally structured condensates have fewer fluorescence peaks and valleys and thus exhibit lower internal voxel intensity SDs (Shiina, 2019; Milano et al, 2024). Using this metric, we found that ER stress reduced the SD of voxel intensity within Me31B-GFP–marked P-bodies by ∼39.96%, indicating a smoother and more homogeneous internal organization (Fig 1G). Together, this data suggests that P-bodies rapidly remodel their morphology and intracondensate organization within 30 min of ER stress onset.

### P-body remodeling during ER stress precedes stress granule assembly

**Figure 2.**
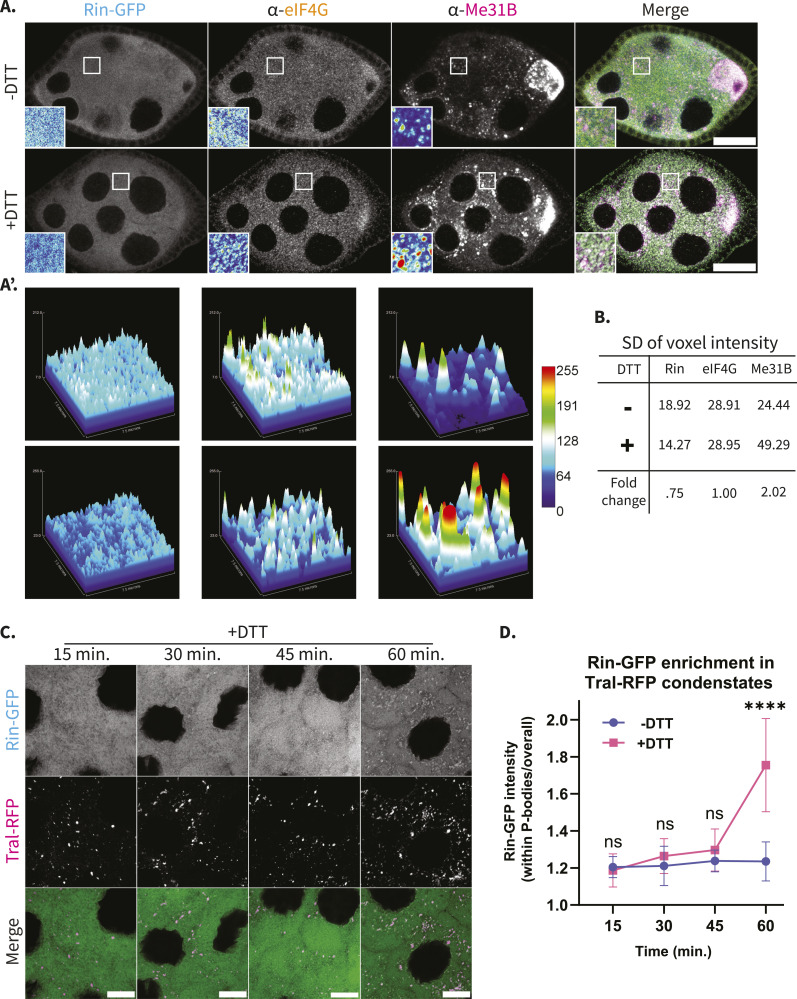
P-body remodeling during ER stress precedes stress granule assembly. **(A)** Endogenously expressed Rin-GFP covisualized with immunolabeled eIF4G and Me31B in egg chambers incubated ex vivo in Schneider’s media alone (−DTT) or in Schneider’s media supplemented with DTT (+DTT). Images are XY projections of 5 optical Z-slices of 0.3 µm. Scale bars are 50 µm. **(A’)** 3D intensity maps of selected regions of interest in (A). **(B)** SD of voxel intensity of regions of interest in (A’). **(C)** Covisualization of endogenously expressed Rin-GFP and Tral-RFP in +DTT-incubated egg chambers over the course of 60 min. Images are XY projections of 5 optical Z-slices of 0.3 µm. Scale bars are 20 µm. **(D)** Quantification of Rin-GFP enrichment within Tral-RFP–labeled P-bodies in −DTT- and +DTT-incubated egg chambers over the course of 60 min (n = 20). For all plots, each data point represents the average value of all P-bodies detected in an image. Significance was assessed using Mann–Whitney statistical tests. Error bars represent the SD. ****P < .0001.

Because P-bodies share multiple components and biophysical properties with stress granules, we next asked whether the observed changes in P-body morphology were secondary to stress granule formation. To address this, we examined egg chambers expressing endogenously tagged Rasputin (Rin)-GFP and performed immunostaining for eIF4G, both *bona fide* stress granule markers (Fig 2A; Buddika et al, 2020). Notably, we did not detect condensation of either Rin-GFP or eIF4G after 30 min of DTT treatment.

To quantitatively assess potential changes in protein distribution, we measured the SD of voxel intensity within defined regions of interest, enabling comparison of spatial heterogeneity across conditions (Fig 2A′). Consistent with our qualitative observations, eIF4G signal distribution remained unchanged between control and DTT-treated egg chambers. Similarly, Rin-GFP signal SD exhibited a modest decrease to 0.75-fold relative to the control. In contrast, Me31B signal SD increased by ∼2.02-fold under DTT treatment, consistent with the pronounced morphological changes observed in P-bodies (Fig 2B). Together, this data indicates that ER stress induces early remodeling of P-bodies in the absence of detectable stress granule assembly.

**Figure S2.**
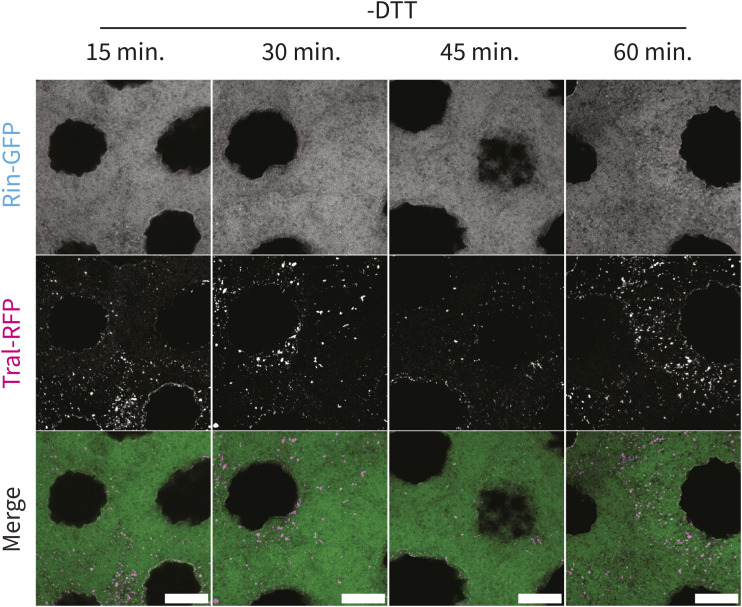
P-body remodeling during ER stress precedes stress granule assembly. Covisualization of endogenously expressed Rin-GFP and Tral-RFP in −DTT-incubated egg chambers over a 60-min time course. Images are XY projections of 5 optical Z-slices of 0.3 µm. Scale bars are 20 µm.

We next sought to define the temporal relationship between P-body remodeling and stress granule formation after ER stress. Previous studies have suggested that stress granules can nucleate from preexisting P-bodies (Buchan et al, 2008). To test whether this occurs under ER stress conditions, we performed a time-course analysis covisualizing Tral-RFP and Rin-GFP (Figs 2C and S2). Rin-GFP did not exhibit increased recruitment to Tral-positive condensates at early time points, and enrichment was only observed after 60 min of ER stress induction, at which point Rin-GFP association increased by ∼42.01% (Fig 2D). Collectively, these results demonstrate that P-bodies undergo rapid morphological and organizational remodeling during the early phase of ER stress, preceding the detectable assembly of stress granules.

### ER stress leads to changes in P-body composition

**Figure 3.**
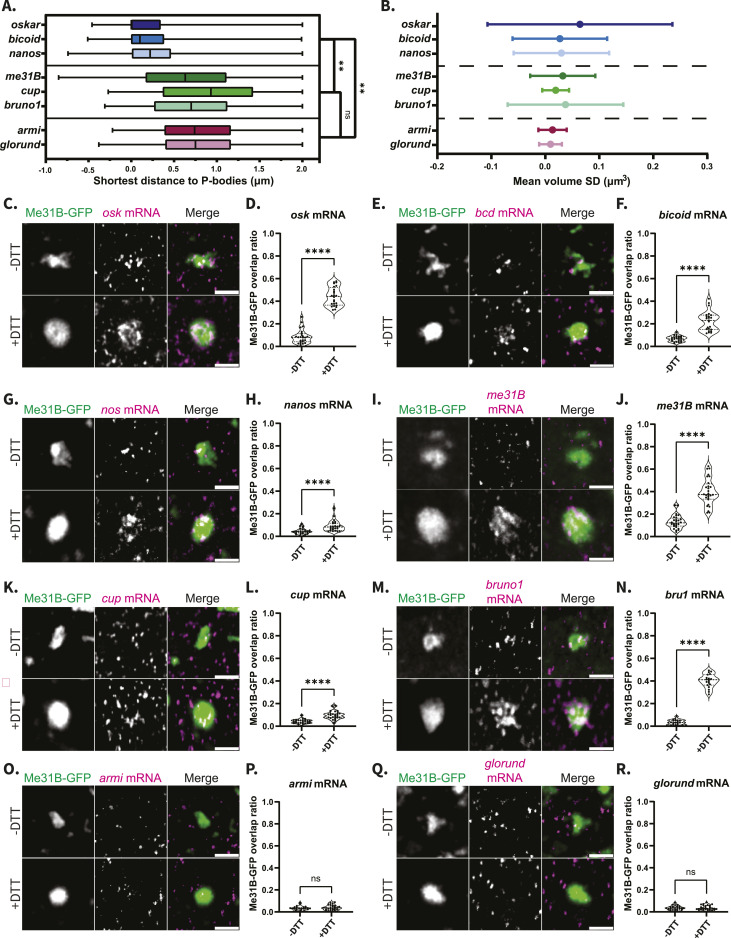
ER stress leads to changes in P-body composition. **(A)** Comparisons between the shortest distance to P-body calculations for three distinct classes of mRNA (shades of blue = maternal mRNAs; shades of green = P-body component mRNAs; shades of pink = non-P-body–associated mRNAs) (n = 3 images; all mRNA particles are represented from each image). Significance was assessed with nested one-way ANOVA. **(B)** Representation of the SD of mRNA particle volume for each of the mRNA classes listed in (A) (n = 3 images; all mRNA particles are represented from each image). **(C)** Covisualization of endogenous Me31B-GFP with *oskar* mRNA labeled with smFISH probes. **(D)** Overlap ratio calculations comparing the amount of Me31B-GFP condensates that overlap with *oskar* mRNA (n = 24). **(E, F)** Same as (C, D) for *bicoid* mRNA (n = 23). **(G, H)** Same as (C, D) for *nanos* mRNA (n = 24). **(I, J)** Same as (C, D) for *me31B* mRNA (n = 24). **(K, L)** Same as (C, D) for *cup* mRNA (n = 24). **(M, N)** Same as (C, D) for *bruno1* mRNA (n = 19). **(O, P)** Same as (C, D) for *armi* mRNA (n = 24). **(Q, R)** Same as (C, D) for *glorund* mRNA (n = 24). All images are XY projections of 5 optical Z-slices of 0.3 µm. Scale bars are 2 µm. For all overlap ratio plots, each data point represents the average value of all Me31B-GFP condensates detected in an image. Significance was assessed using Mann–Whitney statistical tests. Error bars represent the SD. ****P < .0001.

**Figure S3.**
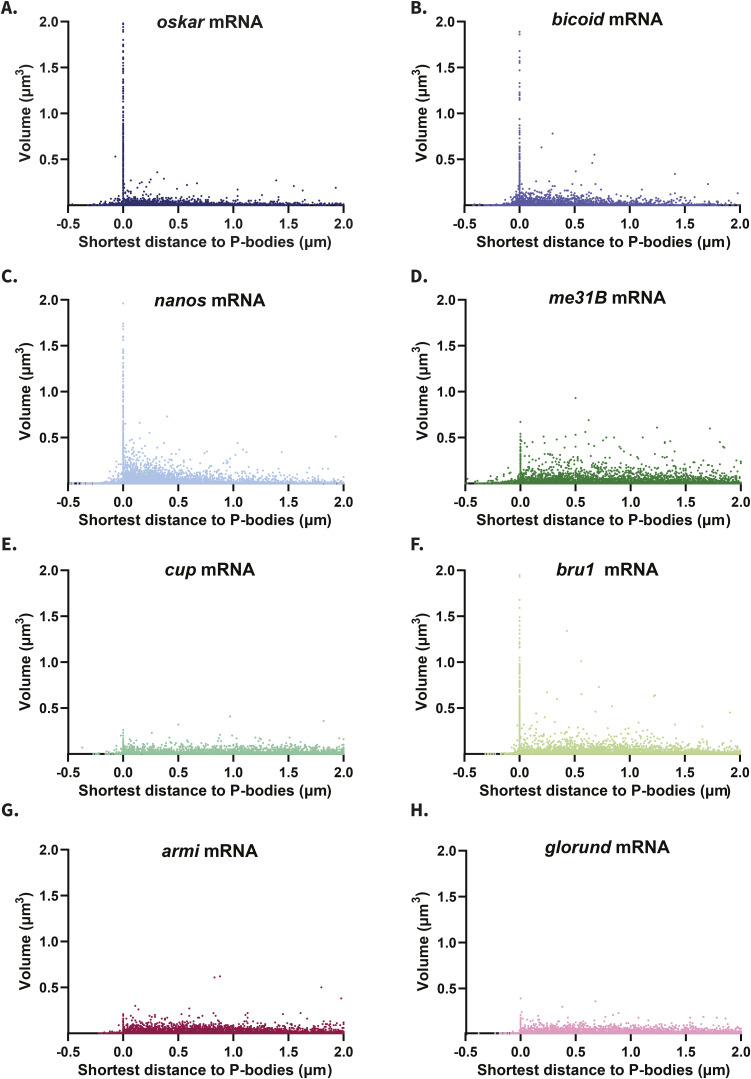
ER stress leads to changes in P-body composition. **(A, B, C, D, E, F, G, H)** Vantage plots showing the shortest distance from respective mRNA particles to a Me31B-labeled P-body on the x-axis and showing the volume of each mRNA particle on the y-axis (n = 3 images; all mRNA particles are represented from each image). **(A)** *oskar* mRNA. **(B)** *bicoid* mRNA. **(C)** *nanos* mRNA. **(D)** *me31B* mRNA. **(E)** *cup* mRNA. **(F)** *bruno1* mRNA. **(G)** *armi* mRNA. **(H)** *glorund* mRNA.

Previous studies have indicated that changes in P-body intracondensate organization can lead to a change in P-body composition (Sankaranarayanan et al, 2021; Milano et al, 2026). As we observed clear changes in P-body organization within 30 min of ER stress, we next asked whether the mRNA composition of P-bodies also shifted within this time frame. We chose to explore three distinct classes of mRNAs in our study: (1) maternal mRNAs that normally localize to P-bodies (*oskar, bicoid,* and *nanos)*, (2) the mRNAs of P-body protein components (*me31B, cup,* and *bruno1*), and (3) non-post-transcriptionally regulated mRNAs (*armi* and *glorund*). To determine the localization of each mRNA in our tissue, we created a grouped comparison of the three classes by generating a series of Vantage plots using Imaris software (Fig 3A)*.* These plots allow for the quantification of mRNA partitioning into P-bodies (puncta with the shortest distances to P-bodies of 0 μm or less localize inside, whereas shortest distances larger than 0 μm localize outside P-bodies). Maternal mRNAs showed a much smaller average shortest distance to P-bodies than either other class. This was expected as these mRNAs are known to localize to P-bodies, whereas the other two classes of mRNAs have not been shown to display this type of localization. Next, we plotted mRNA punctum volume against the shortest distance to Me31B-GFP–marked P-bodies (Fig S3A–H). Notably, mRNA particles with shorter distances to P-bodies displayed substantially larger volumes than particles with longer shortest distances to P-bodies, indicating the accumulation of multiple mRNA molecules within these condensates.

In addition, we calculated the SD of mRNA punctum volume for each species to compare the propensity of each mRNA to exist as multicopy mRNPs. mRNAs that exist primarily as single copies will have low volume SDs, whereas mRNAs that exist in the cytoplasm both as single copies and as multicopy puncta will have larger volume SDs (Fig 3B). Unsurprisingly, all maternal mRNAs displayed volume SDs much larger than the non-P-body mRNAs. However, it was of note that the P-body component mRNAs seemed to behave independent of their class. *me31B* and *bruno1* mRNAs showed SDs much more comparable to the maternal mRNAs, whereas *cup* mRNA showed a volume SD much closer to the non-P-body–associated mRNAs. This may indicate that these mRNAs, while they are not putative P-body–localized transcripts based on the literature, do display a propensity to form large multicopy mRNPs.

Based on these classifications, we first examined the localization of maternal mRNAs after 30 min of ER stress. Under basal conditions, P-bodies contain several *oskar* mRNA molecules; however, after DTT treatment, P-bodies appeared markedly enriched in *oskar* mRNA (Fig 3C). Because the density of *oskar* mRNA puncta precluded reliable quantification of individual transcripts, we instead quantified the overlap volume ratio between Me31B-GFP–labeled P-bodies and *oskar* mRNA. Using this approach, we observed an ∼4.99-fold increase in Me31B-GFP signal overlapping with *oskar* mRNA after ER stress (Fig 3D). We next examined two additional maternal transcripts, *bicoid* and *nanos*, and observed similar trends. The ER stress induced an ∼3.33-fold increase in overlap between Me31B-GFP and *bicoid* mRNA and an ∼1.95-fold increase for *nanos* mRNA (Fig 3E–H). Together, these results indicate that ER stress alters P-body composition by increasing the amount of maternal mRNAs enriched within these granules.

We next examined mRNAs encoding P-body components. Visualization of *me31B* mRNA after ER stress revealed a similar enrichment within P-bodies, with an ∼3.03-fold increase in overlap volume ratio between *me31B* mRNA and Me31B-GFP–labeled P-bodies (Fig 3I and J). This observation was notable because *me31B* mRNA is not typically considered a frequent P-body client based on prior studies and our own analysis (Fig 3A). We then analyzed *cup* mRNA, which appeared largely excluded from P-bodies under basal conditions. After DTT-induced ER stress, however, *cup* mRNA localized to P-bodies, exhibiting an ∼2.23-fold increase in overlap volume ratio (Fig 3K and L). Finally, we examined *bruno1* mRNA and observed a pronounced enrichment within P-bodies, with an ∼11.49-fold increase in overlap ratio after ER stress (Fig 3M and N). The Bruno 1 protein is a well-characterized P-body–associated RNA-binding protein that binds target mRNAs and recruits Cup to transcripts, although its own transcript has not previously been reported as a strong P-body client (Nakamura et al, 2004). Notably, analysis of mRNA punctum volume SD revealed that *bruno1* mRNA exhibited a distribution similar to that of maternal transcripts known to localize to P-bodies (Figs 3B and S3F). Together, these results suggest that ER stress promotes recruitment of mRNAs encoding P-body components to P-bodies, despite these transcripts not typically behaving as canonical P-body clients.

To determine whether this recruitment was specific, we next examined two transcripts not associated with P-bodies, *armi* and *glorund*. Both genes encode non-P-body–associated mRNAs that are broadly expressed during oogenesis (Tomari et al, 2004; Kalifa et al, 2009). Consistent with previous reports and our Vantage plot analysis, these transcripts displayed large average distances from P-bodies and relatively small SD in puncta volume, indicating that they predominantly exist as single-copy puncta (Fig 3A and B). In contrast to the transcripts described above, ER stress did not alter the association of *armi* mRNA (Fig 3O and P) or *glorund* mRNA (Fig 3Q and R) with P-bodies. These observations indicate that ER stress does not drive indiscriminate transcript recruitment, but instead promotes the selective enrichment of specific classes of mRNAs within P-bodies.

### mRNAs associated with P-bodies during ER stress are protected from degradation

ER stress activates the endoribonuclease Ire1, which initiates both XBP1 splicing and regulated Ire1-dependent decay (RIDD), a pathway that promotes widespread mRNA degradation to reduce ER load. In *D. melanogaster*, RIDD has been shown to be largely promiscuous, with transcript susceptibility to degradation governed primarily by proximity to the ER membrane rather than sequence features (Gaddam et al, 2013). Because nurse cells are highly enriched in ER membranes, this raised the possibility that many transcripts may be vulnerable to degradation during ER stress.

**Figure 4.**
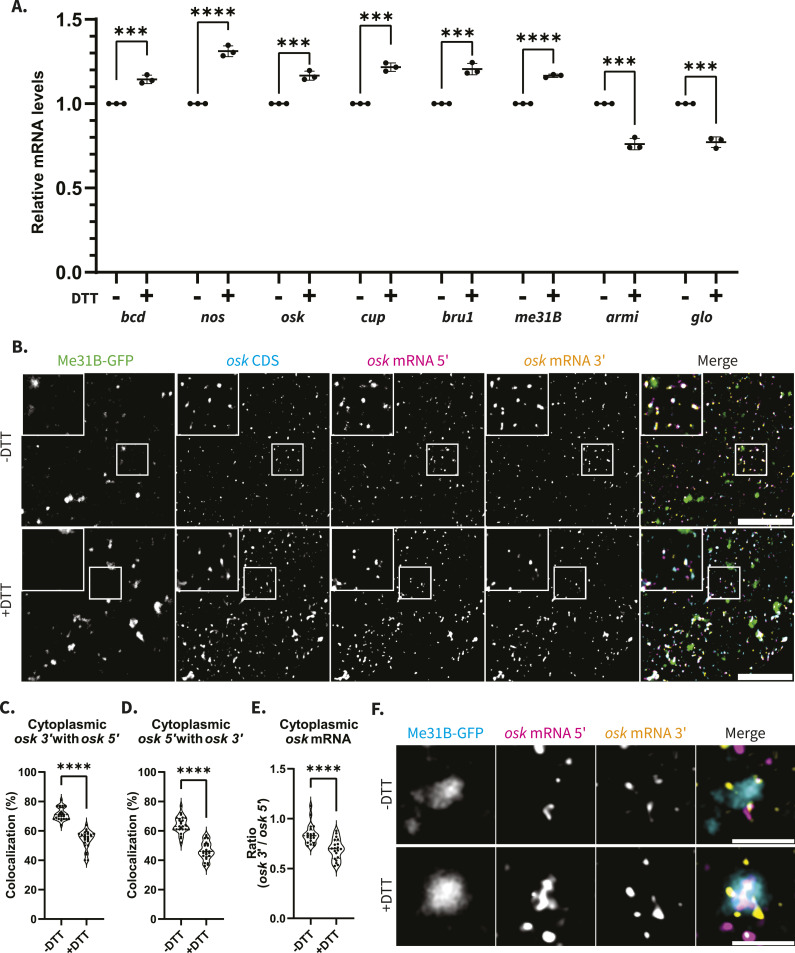
mRNAs associated with P-bodies during ER stress are protected from degradation. **(A)** RT–qPCR quantification of 8 mRNAs in −DTT- and +DTT-incubated egg chambers. Significance was calculated with Welch’s *t* test (n = 3, biological replicates). **(B)** Endogenously expressed Me31B-GFP covisualized with smFISH-labeled oskar with differentially labeled CDS 5′ region and 3′ region. Images are XY projections of 5 optical Z-slices of 0.3 µm. Scale bars are 10 µm. **(C)** Colocalization analysis between the *osk* 3′– and *osk* 5′–labeled puncta. Only puncta that also colocalized with the CDS were used in the calculations (n = 25). **(D)** Colocalization analysis between the *osk* 5′– and *osk* 3′–labeled puncta. Only puncta that also colocalized with the CDS were used in the calculations (n = 25). **(E)** Ratio of total *osk* 3′ puncta over total *osk* 5′ puncta (n = 20). **(F)** Covisualization of endogenous Me31B-GFP with smFISH-labeled *osk* 3′ and *osk* 5′ regions via STED. Images are XY projections of 5 optical Z-slices of 0.22 µm. The scale bar is 2 µm. For all plots based on imaging, each data point represents the average value of all mRNAs detected in an image. Significance was assessed using Mann–Whitney statistical tests. Error bars represent the SD. ***P < .001, ****P < .0001.

Given the selective recruitment of specific mRNAs to enlarged P-bodies after ER stress, we asked whether P-body association functions to protect transcripts from degradation. To broadly assess transcript fate, we quantified the abundance of the eight mRNAs by RT–qPCR after 30 min of ER stress. Notably, the two non-P-body–associated mRNAs, *armi* and *glorund*, showed a significant decrease in abundance (an ∼23.92% decrease for *armi* mRNA and an ∼22.84% decrease for *glorund* mRNA), indicating that ER stress is accompanied by mRNA degradation in nurse cells (Fig 4A). In contrast, all mRNAs that were recruited to P-bodies during ER stress, including maternal mRNAs and P-body component mRNAs, showed increased overall abundance (*bcd* mRNA ∼14.46%, *nos* mRNA ∼31.18%, *osk* mRNA ∼16.63%, *cup* mRNA ∼21.72%, *bru1* mRNA ∼20.56%, and *me31B* mRNA ∼16.57%) (Fig 4A).

**Figure S4.**
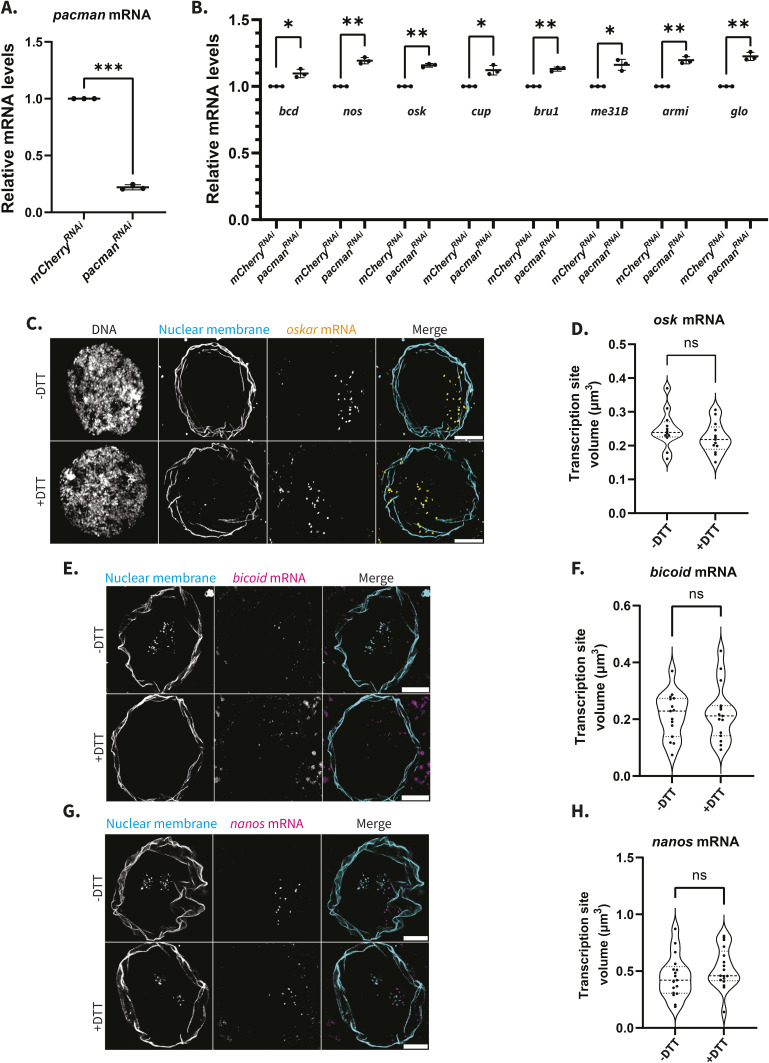
mRNAs associated with P-bodies during ER stress are protected from degradation. **(A)** RT–qPCR quantification of *pacman* mRNAs in *mCherry*^*RNAi*^ and *pacman*^*RNAi*^ egg chambers. Significance was calculated with Welch’s *t* test (n = 3). **(B)** RT–qPCR quantification of 8 mRNAs in *mCherry*^*RNAi*^ and *pacman*^*RNAi*^ egg chambers. Significance was calculated with Welch’s *t* test (n = 3). **(C)** Covisualization of *oskar* mRNA labeled with smFISH probes with DAPI-labeled DNA and nuclear membrane stained with wheat agglutinin in −DTT- and +DTT-incubated egg chambers. Images are XY projections of 15 optical Z-slices of 0.3 µm. Scale bars are 10 µm. **(D)** Volume quantification of the *oskar* transcription sites in (C) (n = 30). **(E)** Covisualization of *bicoid* mRNA labeled with smFISH probes with the nuclear membrane stained with wheat agglutinin in −DTT- and +DTT-incubated egg chambers. Images are XY projections of 15 optical Z-slices of 0.3 µm. Scale bars are 10 µm. **(F)** Volume quantification of the *bicoid* transcription sites in (E) (n = 15). **(G)** Covisualization of *nanos* mRNA labeled with smFISH probes with the nuclear membrane stained with wheat agglutinin in −DTT- and +DTT-incubated egg chambers. Images are XY projections of 15 optical Z-slices of 0.3 µm. Scale bars are 10 µm. **(H)** Volume quantification of the *nanos* transcription sites in (G) (n = 17). For all plots, each data point represents the average value of all transcription sites detected in an image. Significance was assessed using Mann–Whitney statistical tests. Error bars represent the SD. *P < .1, **P < .01, ****P < .0001.

The surprising finding that transcript levels increased above control levels cannot be explained only by protection from degradation associated with ER stress as this does not occur under basal conditions. We hypothesized that the increase in the level of these mRNAs was due to enrichment of the transcripts within P-bodies where they would be protected from all background degradation, even those which they would be exposed to under basal conditions. Although these transcripts are not canonical targets of the mRNA decay machinery, it is possible that a fraction undergoes low-level “leaky” degradation under steady-state conditions. To address this at basal conditions, we evaluated the level of these mRNAs in the RNAi knockdown background of the key 5′ to 3′ exonuclease enzyme Pacman (Xrn1) to reduce the “leaky” degradation. *pacman*^*RNAi*^ was induced using the UAS/GAL4 system, and the efficiency of the knockdown was confirmed via RT–qPCR (Fig S4A). Consistent with our hypothesis, all the transcripts increased in abundance in this background, suggesting that under basal conditions, there is low-grade leaky degradation of some transcripts (Fig S4B).

To determine whether the increased levels of the P-body–associated mRNAs indeed resulted from protection from degradation or were possibly due to elevated transcription, we assessed transcriptional activity by measuring the volume of nascent transcription sites. Analysis of *oskar* transcription revealed no change in transcription site volume after ER stress (Fig S4C and D). Similarly, transcription site volumes for *bicoid* and *nanos* were unchanged, indicating that increased maternal mRNA abundance does not arise from increased transcription, but instead results from increased protection from degradation (Fig S4E–H).

We next asked whether during ER stress P-body–associated maternal mRNAs are more protected from degradation versus cytoplasmic maternal mRNAs that are not within P-bodies. To assess cytoplasmic mRNA stability, we employed dual-color single-molecule fluorescence in situ hybridization (smFISH) using probe sets differentially targeting the 5′ and 3′ ends of *oskar* mRNA and excluded from our analysis any signal that was colocalized with Me31B-GFP–labeled P-bodies (Fig 4B). To ensure that all 5′- and 3′-end signals were representative of true *oskar* transcripts, we only quantified puncta that colocalized with the separately labeled *oskar* mRNA coding sequence. Protected or compact mRNAs exhibit reduced spatial separation between their 5′ and 3′ ends, resulting in increased probe colocalization (Adivarahan et al, 2018; Khong & Parker, 2018). After ER stress, cytoplasmic *oskar* mRNA showed an ∼24.43% decrease in 3′:5′ colocalization and an ∼27.52% decrease in 5′:3′ colocalization compared with control conditions, indicating decreased mRNA compaction and protection (Fig 4C and D). Consistent with this interpretation, the ratio of 3′ to 5′ puncta decreased by ∼17.96% after ER stress, suggesting increased transcript degradation as there were more 5′ puncta than 3′ puncta in the cytoplasm (Fig 4E). Together, these data indicate that maternal mRNAs that are not recruited to P-bodies during ER stress are more susceptible to cytoplasmic degradation. After ER stress, a greater fraction of *oskar* transcripts localizes to P-bodies, accompanied by an overall increase in total *oskar* abundance. Despite this increase, we observe elevated degradation of *oskar* transcripts that remain outside of P-bodies. These findings support a model in which P-bodies function as protective compartments that selectively shield mRNAs from enhanced cytoplasmic decay during ER stress.

To further investigate the organization of *oskar* mRNA within P-bodies during ER stress, we used these same dual-color smFISH probes coupled with STED super-resolution microscopy. Notably, under control conditions *oskar* 5′ and 3′ ends were colocalized within P-bodies but, after ER stress, the 5′ and 3′ ends segregated with the *oskar* 5′ ends localizing to the center of P-bodies and *oskar* 3′ ends localizing to the periphery of the condensates (Fig 4F). Interestingly, this is consistent with the previous work that showed that granule state can affect mRNA intracondensate organization (Ramat et al, 2024). Together, this data suggests that ER stress leads to a change in *oskar* mRNA stability with cytoplasmic particles being more prone to degradation and P-body–associated particles reorganizing within the granules.

### Bruno 1 is up-regulated during ER stress and is required for P-body remodeling

**Figure S5.**
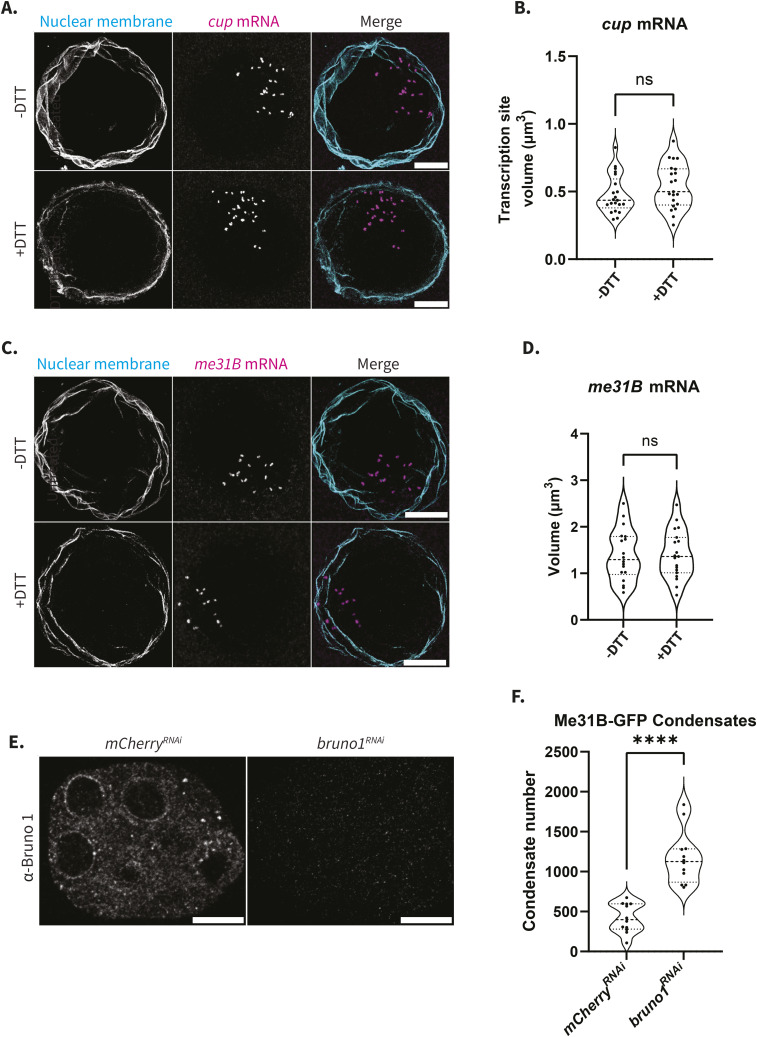
Bruno 1 is up-regulated during ER stress and is required for P-body remodeling. **(A)** Covisualization of *cup* mRNA labeled with smFISH probes with the nuclear membrane stained with wheat agglutinin in −DTT- and +DTT-incubated egg chambers. Images are XY projections of 15 optical Z-slices of 0.3 µm. Scale bars are 10 µm. **(B)** Volume quantification of the *cup* transcription sites in (A) (n = 21). **(C)** Covisualization of *me31B* mRNA labeled with smFISH probes with the nuclear membrane stained with wheat agglutinin in −DTT- and +DTT-incubated egg chambers. Images are XY projections of 15 optical Z-slices of 0.3 µm. Scale bars are 10 µm. **(D)** Volume quantification of the *me31B* transcription sites in (C) (n = 18). **(E)** Visualization of immunolabeled Bruno 1 in *mCherry*^*RNAi*^ and *bruno1*^*RNAi*^ egg chambers. Images are XY projections of 5 optical Z-slices of 0.3 µm. Scale bars are 100 µm. **(F)** Quantification of the overall Me31B-GFP condensate number in *mCherry*^*RNAi*^ and *bruno1*^*RNAi*^ egg chambers (n = 12). For all plots, each data point represents the average value of all transcription sites detected in an image. Significance was assessed using Mann–Whitney statistical tests. Error bars represent the SD. ****P < .0001.

**Figure 5.**
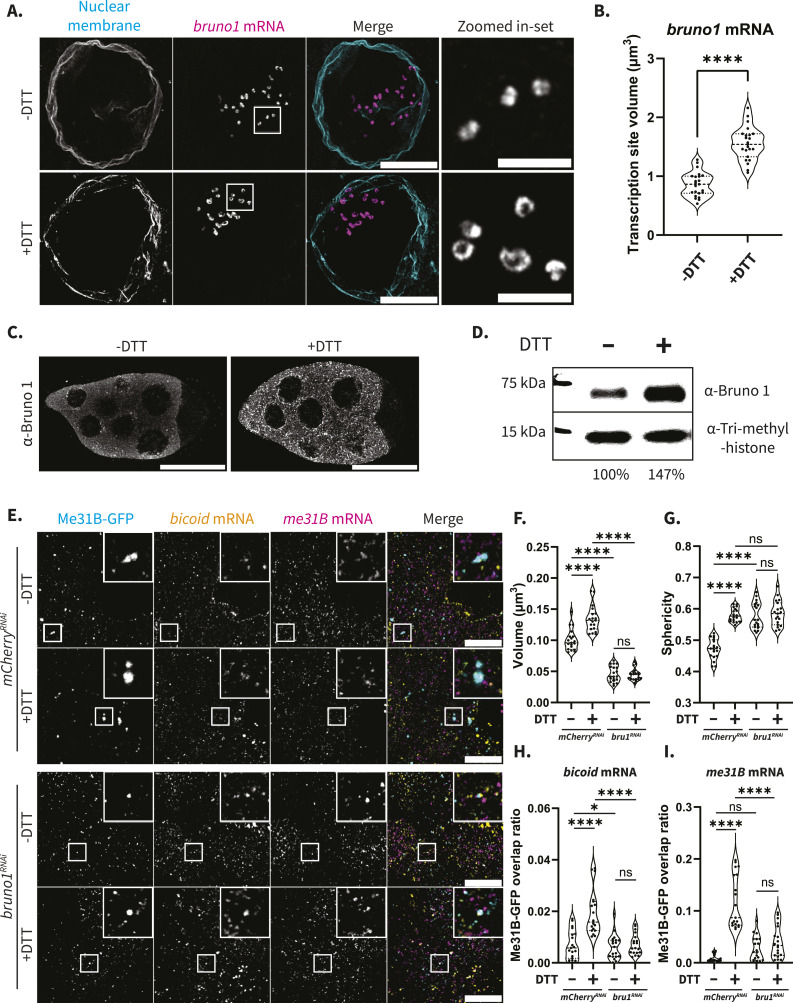
Bruno 1 is up-regulated during ER stress and is required for P-body remodeling. **(A)** Covisualization of *bruno1* mRNA labeled with smFISH probes and the nuclear membrane visualized with wheat agglutinin staining. Images are XY projections of 15 optical Z-slices of 0.3 µm. Scale bars are 10 µm. **(B)** Quantification of *bruno1* transcription site volume in −DTT- and +DTT-incubated egg chambers (n = 23). **(C)** Visualization of immunolabeled Bruno 1 in −DTT- and +DTT-incubated egg chambers. Images are XY projections of 5 optical Z-slices of 0.3 µm. Scale bars are 100 µm. **(D)** Western blot analysis of Bruno 1 in −DTT- and +DTT-incubated egg chambers (Tri-methyl-Histone—loading control). **(E)** Covisualization of endogenously tagged Me31B-GFP with *bicoid* and *me31B* mRNA labeled with smFISH probes in −DTT- and +DTT-incubated *mCherry*^*RNAi*^ (control) and *bruno1*^*RNAi*^ egg chambers. Images are XY projections of 5 optical Z-slices of 0.3 µm. Scale bars are 20 µm. **(F)** Volume quantifications for Me31B-GFP-labeled condensates in (E) (n = 19). **(G)** Sphericity quantifications for Me31B-GFP–labeled condensates in (E) (n = 19). **(H)** Overlap ratio calculations comparing the amount of Me31B-GFP condensates that overlap with *bicoid* mRNA in (E) (n = 19). **(I)** Overlap ratio calculations comparing the amount of Me31B-GFP condensates that overlap with *me31B* mRNA in (E) (n = 19). For all plots, each data point represents the average value of all mRNAs and Me31B-GFP–labeled condensates detected in an image. Significance was assessed using Mann–Whitney statistical tests. Error bars represent the SD. ****P < .0001.

To determine whether increased abundance of P-body component mRNAs after ER stress was driven by transcriptional up-regulation, we quantified transcription site volumes for *me31B*, *cup*, and *bruno1*. Transcription site analysis revealed no significant change in *me31B* or *cup* transcription after ER stress (Fig S5A–D). Surprisingly, in contrast, *bruno1* transcription site volume increased by ∼80.12% after 30 min of ER stress, suggesting an overall increase in *bruno1* transcription (Fig 5A and B). Furthermore, increased *bruno1* transcription resulted in elevated Bruno 1 protein levels detected via immunostaining after ER stress (Fig 5C). Consistent with this observation, Western blot analysis revealed an ∼47% increase in total Bruno 1 protein levels after 30 min of ER stress (Fig 5D). Together, this data demonstrates that ER stress selectively induces *bruno1* transcription, resulting in increased Bruno 1 protein expression.

Because Bruno 1 is a core P-body component that binds directly to mRNA and interacts with other P-body proteins, including Cup, we next asked whether Bruno 1 contributes to our previously observed ER stress–induced changes in P-body morphology (Kim-Ha et al, 1995; Nakamura et al, 2004; Bayer et al, 2023). To test this, we performed an RNAi-facilitated knockdown of Bruno 1 using the UAS/GAL4 system and visualized P-bodies using endogenously expressed Me31B-GFP (Fig 5E). RNAi efficiency was confirmed via immunostaining (Fig S5E). Because Bruno 1 knockdown egg chambers arrest at early developmental stages, we re-evaluated ER stress effects at stage 3 of oogenesis, as all our prior quantifications focused on mid-stage egg chambers.

Under basal conditions, Me31B-GFP–marked P-bodies were ∼55.28% smaller in the *bruno1*^*RNAi*^ background compared with control (*mCherry*^*RNAi*^) egg chambers, and ∼22.38% more spherical, indicating that Bruno 1 contributes to P-body morphology even in the absence of ER stress (Fig 5F and G). Accompanying this reduction in condensate volume, we also observed an ∼2.77-fold increase in the overall number of P-bodies in the *bruno1*^*RNAi*^ egg chambers compared with controls (Fig S5F). Although we could not assess the overall level of the Me31B protein via Western blot analysis, as Bruno 1 knockdown ovaries do not develop past stage 4 of oogenesis and it is difficult to accurately compare protein levels at specific stages, immunofluorescence imaging suggests that overall Me31B levels remain the same (Fig 5E). Together, this data indicates that in the absence of Bruno 1, P-bodies reorganize into an increased number of smaller, more spherical condensates.

Strikingly, whereas P-bodies in control egg chambers increased in volume by ∼22.87% and sphericity by ∼33.45% after ER stress, P-bodies in the Bruno 1 knockdown background showed no detectable change in morphology between basal and ER stress conditions (Fig 5E–G). This indicates that Bruno 1 is necessary for ER stress–induced P-body enlargement and confirms that P-bodies respond to ER stress at multiple stages of development.

We next asked whether Bruno 1 is also required for functional changes in P-body composition during ER stress. To assess this, we quantified the overlap volume ratio between Me31B-GFP–labeled P-bodies and representative P-body–associated mRNAs (*bicoid* and *me31B*) to assess mRNA association with P-bodies. We chose not to use *oskar* as our maternal mRNA representative as Bruno 1 has been shown to directly dictate its post-transcriptional regulation and this may make it difficult to parse changes at the condensate level (Kim-Ha et al, 1995; Snee et al, 2008). In control egg chambers, ER stress led to an ∼14.42-fold increase in Me31B-GFP overlap ratio with *bicoid* mRNA and an ∼2.77-fold increase in overlap ratio with *me31B* mRNA (Fig 5E). In contrast, in the *bruno1*^*RNAi*^ background, ER stress failed to significantly increase the enrichment of either mRNA in P-bodies (Fig 5H and I). Thus, Bruno 1 is required, not only for ER stress–induced changes in P-body morphology, but also for the consequent recruitment of mRNAs to P-bodies during the ER stress response.

### ATF4 acts upstream of Bruno 1 during ER stress response

**Figure 6.**
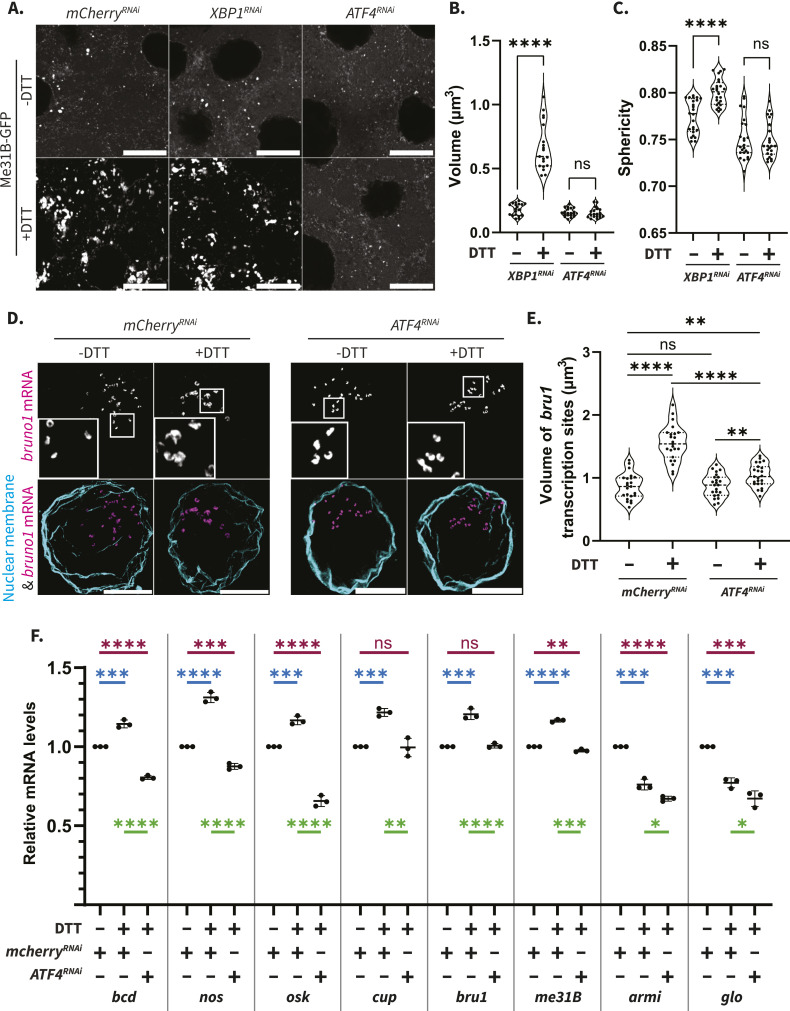
ATF4 acts upstream of Bruno 1 during ER stress response. **(A)** Endogenously labeled Me31B-GFP visualized in *mCherry*^*RNAi*^*, XBP1*^*RNAi*^*,* and *ATF4/crc*^*RNAi*^ egg chambers incubated in −DTT and +DTT. Images are XY projections of 5 optical Z-slices of 0.3 µm. Scale bars are 20 µm. **(B)** Volume quantifications for Me31B-GFP condensates in (A) (n = 25). **(C)** Sphericity quantifications for Me31B-GFP condensates in (A) (n = 24). **(D)** *bruno1* mRNA labeled with smFISH probes visualized with wheat agglutinin nuclear membrane stain in *mCherry*^*RNAi*^ and *ATF4/crc*^*RNAi*^ egg chambers incubated in −DTT and +DTT. Images are XY projections of 15 optical Z-slices of 0.3 µm. Scale bars are 10 µm. **(E)** Quantification of *bruno1* transcription site volume in (D) (n = 20). **(F)** RT–qPCR quantification of 8 mRNAs in *mCherry*^*RNAi*^ and *ATF4/crc*^*RNAi*^ egg chambers incubated in −DTT and +DTT. Significance was calculated with Welch’s *t* test (n = 3). For all plots based on imaging, each data point represents the average value of all Me31B-GFP–labeled condensates or mRNAs detected in an image. Significance was assessed using Mann–Whitney statistical tests. Error bars represent the SD. **P < .01, ***P < .001, ****P < .0001.

**Figure S6.**
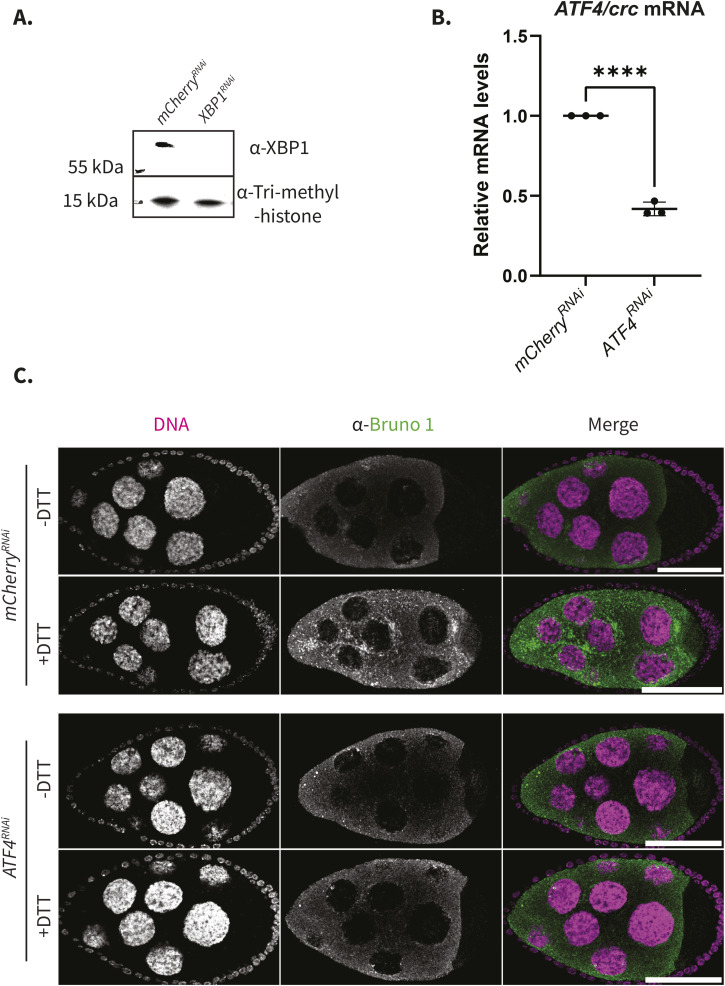
ATF4 acts upstream of Bruno 1 during ER stress response. **(A)** Western blot analysis of XBP1 in *mCherry*^*RNAi*^ and *XBP1*^*RNAi*^ egg chambers (Tri-methyl-Histone—loading control). **(B)** RT–qPCR quantification of *ATF4/crc* mRNA in *mCherry*^*RNAi*^ and *ATF4/crc*^*RNAi*^ egg chambers. Significance was calculated with Welch’s *t* test (n = 3). **(C)** Covisualization of immunolabeled Bruno and DAPI-labeled DNA in *mCherry*^*RNAi*^ and *ATF4/crc*^*RNAi*^ egg chambers incubated in −DTT and +DTT. Images are XY projections of 5 optical Z-slices of 0.3 µm. Scale bars are 100 µm. Error bars represent the SD. ****P < .0001.

Because we found that increased Bruno 1 levels are required for ER stress–induced P-body remodeling, we sought to decipher the underlying mechanism of how ER stress signaling leads to *bruno1* up-regulation. The UPR activates three major transcriptional arms leading to the nuclear localization of three transcription factors, XBP1, ATF4, and ATF6 (Walter & Ron, 2011). These three proteins are highly conserved, and in *D. melanogaster*, the ATF4 gene name is *cryptocephal* (*crc*) (Hewes et al, 2000). To determine which pathway is required for P-body remodeling, we examined Me31B-GFP–labeled P-bodies in *XBP1*^*RNAi*^ and *ATF4*/*crc*^*RNAi*^ backgrounds (Fig 6A). Knockdown efficiency was confirmed via Western blotting for *XBP1*^*RNAi*^ and RT–qPCR for *ATF4/crc*^*RNAi*^ (Fig S6A and B).

In control egg chambers, ER stress induced robust enlargement and increased sphericity of Me31B-GFP–marked P-bodies after 30 min (Fig 6B and C). A similar response was observed in the *XBP1*^*RNAi*^ background with an ∼4.25-fold increase in P-body volume and an ∼3.35% increase in P-body sphericity, indicating that XBP1 is dispensable for ER stress–induced P-body remodeling (Fig 6B and C). In contrast, P-bodies in the *ATF4/crc*^*RNAi*^ background failed to undergo any detectable morphological changes after ER stress, phenocopying the *bruno1*^*RNAi*^ egg chambers (Fig 6B and C). These results suggest that P-body remodeling occurs downstream of the ATF4 arm of the ER stress response.

To test whether ATF4 is required for ER stress–induced increased *bruno1* transcription, we quantified *bruno1* transcription sites in the *ATF4*/*crc*^*RNAi*^ background. Under basal conditions, *bruno1* transcription was comparable between control and ATF4 knockdown egg chambers. However, after ER stress, *bruno1* transcription increased only modestly, ∼12.63% in the absence of ATF4, compared with ∼81.13% in control egg chambers (Fig 6D and E). Consistent with this result, immunostaining revealed no appreciable increase in Bruno 1 protein levels after ER stress in the *ATF4/crc*^*RNAi*^ background (Fig S6C). Together, this data indicates that ER stress–induced up-regulation of Bruno 1 is largely dependent on ATF4 activity.

Finally, we asked whether loss of ATF4 affected the fate of the eight representative mRNAs during ER stress. To assess this, we quantified transcript abundance after ER stress in the *ATF4*/*crc*^*RNAi*^ background. As in controls, non-P-body–associated mRNA levels decreased after ER stress (*armi* decreased by ∼32.88% and *glorund* decreased by ∼32.83%) (Fig 6F). In contrast, maternal mRNAs that were protected and increased in abundance in control egg chambers instead decreased significantly in the absence of ATF4 (*bicoid* mRNA by ∼19.49%, *nanos* mRNA by ∼12.47%, and *oskar* mRNA by ∼34.36%). Similarly, P-body component mRNAs failed to increase in abundance after ER stress in the *ATF4*/*crc*^*RNAi*^ background (Fig 6F). Thus, loss of ATF4 abolishes both P-body remodeling and selective mRNA protection during ER stress.

### Bruno 1 overexpression is sufficient to drive P-body remodeling and rescues morphology in the ATF4 knockdown background

**Figure S7.**
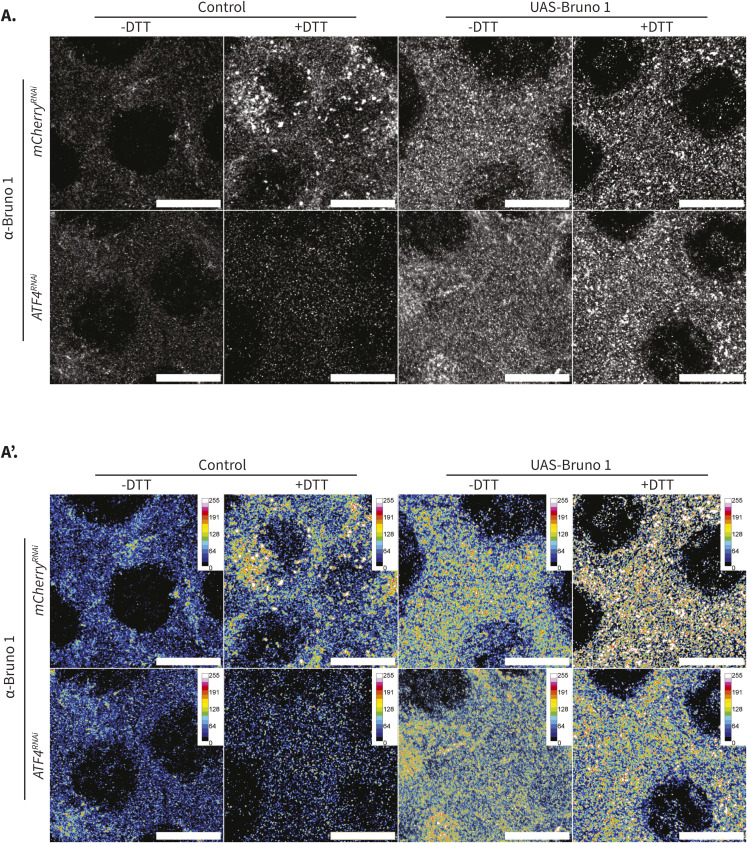
Bruno 1 overexpression is sufficient to drive P-body remodeling and rescues morphology in the ATF4 knockdown background. **(A)** Visualization of Bruno 1 in control and Bruno 1 overexpression backgrounds (UAS-Bruno 1) in *mCherry*^*RNAi*^ and *ATF4/crc*^*RNAi*^ egg chambers incubated in −DTT and +DTT. Images are XY projections of 5 optical Z-slices of 0.3 µm. Scale bars are 20 µm. **(A’)** Intensity heat map of (A).

**Figure 7.**
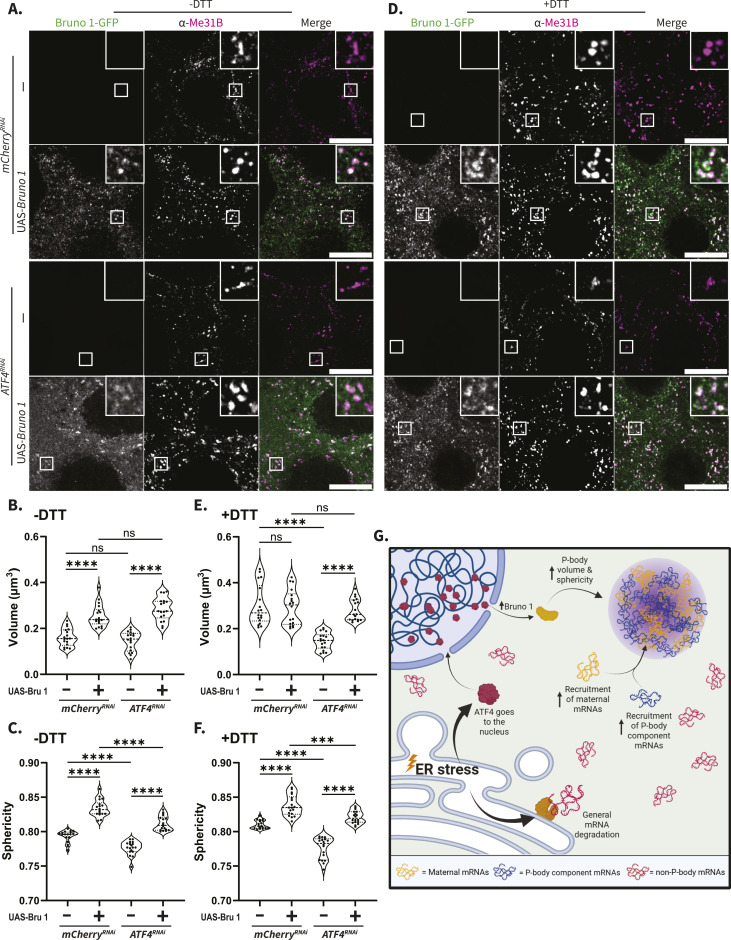
Bruno 1 overexpression is sufficient to drive P-body remodeling and rescues morphology in the ATF4 knockdown background. **(A)** Covisualization of transgenic Bruno 1-GFP and immunolabeled Me31B in *mCherry*^*RNAi*^ and *ATF4/crc*^*RNAi*^ egg chambers incubated in Schneider’s media (−DTT). Images are XY projections of 5 optical Z-slices of 0.3 µm. Scale bars are 20 µm. **(B)** Volume quantifications for Me31B condensates in (A) (n = 18). **(C)** Sphericity quantifications for Me31B condensates in (A) (n = 18). **(D)** Covisualization of transgenic Bruno 1-GFP and immunolabeled Me31B in *mCherry*^*RNAi*^ and *ATF4/crc*^*RNAi*^ egg chambers incubated in Schneider’s media supplemented with DTT (+DTT). Images are XY projections of 5 optical Z-slices of 0.3 µm. Scale bars are 20 µm. **(E)** Volume quantifications for Me31B condensates in (D) (n = 18). **(F)** Sphericity quantifications for Me31B condensates in (D) (n = 18). **(G)** Proposed mechanism of how ER stress triggers P-body response. For all plots, each data point represents the average value of all Me31B-labeled condensates or mRNAs detected in an image. Significance was assessed using Mann–Whitney statistical tests. Error bars represent the SD. ***P < .001, ****P < .0001.

Because loss of ATF4 abolished both ER stress–induced P-body remodeling and Bruno 1 up-regulation, we next asked whether increasing Bruno 1 levels alone is sufficient to restore condensate morphological response after ER stress. To test this, we again used the UAS/GAL4 system to specifically up-regulate transgenic Bruno 1-GFP expression in the female germline. Previous studies have demonstrated that this overexpression line is viable (Nikonova et al, 2024). Immunofluorescence analysis confirmed that this approach resulted in elevated Bruno 1 protein levels (Fig S7A and A′). Under basal conditions, the overexpression of Bruno 1-GFP in *mCherry*^*RNAi*^ control egg chambers led to a marked change in P-body morphology, with Me31B-GFP–labeled P-bodies exhibiting an ∼58.32% increase in volume and an ∼5.12% increase in sphericity (Fig 7A–C). Notably, a similar increase in P-body volume and sphericity was observed in the *ATF4*/*crc*^*RNAi*^ egg chambers, indicating that elevated Bruno 1 levels are sufficient to promote P-body remodeling independent of ATF4 activity.

We next examined whether further increases in Bruno 1 levels could augment P-body remodeling during ER stress (Fig 7D). In the *mCherry*^*RNAi*^ background, Bruno 1 overexpression increased P-body sphericity but did not further increase P-body volume after ER stress, suggesting that P-body volume may be constrained by a threshold level of Bruno 1 beyond which there is no additional change in size (Fig 7E and F). Alternatively, there may be a maximum sustainable P-body volume in this tissue.

In contrast, in the *ATF4*/*crc*^*RNAi*^ egg chambers, Bruno 1 overexpression restored P-body volume to levels comparable to control egg chambers after ER stress and led to an even greater increase in P-body sphericity (Fig 7E and F). Together, these results demonstrate that Bruno 1 up-regulation is sufficient to drive P-body remodeling and can compensate for the loss of ATF4 during the ER stress response.

## Discussion

Cytoplasmic condensates such as P-bodies have long been associated with mRNA repression and decay, yet how their properties are actively regulated during cellular stress remains unclear (Eulalio et al, 2007; Luo et al, 2018; Standart & Weil, 2018). Early work in yeast and mammalian systems established P-bodies as dynamic assemblies whose size and number respond to translational status and stress conditions (Brengues, 2005; Teixeira et al, 2005; Parker & Sheth, 2007). More recent studies have emphasized that P-bodies are compositionally heterogeneous, and exhibit liquid-like behaviors shaped by multivalent RNA:protein interactions (Banani et al, 2016; Sankaranarayanan et al, 2021). Our findings extend this framework by demonstrating that ER stress rapidly remodels P-body morphology and internal organization in *D. melanogaster* nurse cells, revealing P-bodies as early and responsive components of the ER stress response rather than passive by-products of translational repression.

Several studies have suggested that stress granules and P-bodies can exchange components and often respond sequentially to stress, with stress granules typically forming after prolonged translational arrest (Kedersha et al, 2005; Riggs et al, 2020). Consistent with this view, we observe pronounced P-body remodeling within 30 min of ER stress induction, in the absence of detectable stress granule formation. This temporal separation suggests that P-bodies may act as an early buffering compartment during ER stress, potentially stabilizing select transcripts before engagement of later stress granule–mediated responses. Such a model aligns with recent proposals that distinct condensates fulfill temporally and functionally specialized roles during stress adaptation (Kroschwald et al, 2015; Choi et al, 2025).

Mechanistically, our data identifies transcriptional up-regulation of the RNA-binding protein Bruno 1 as a key driver of ER stress–induced P-body remodeling (Fig 7G). Previous work has established Bruno 1 as a translational repressor essential for maternal mRNA regulation during oogenesis and as a component of P-bodies (Webster et al, 1997; Chekulaeva et al, 2006; Bayer et al, 2023). By showing that ER stress induces up-regulation of *bruno1* transcription in an ATF4-dependent manner and that loss of Bruno 1 abolishes P-body compositional remodeling, we uncover a link between stress-responsive transcriptional programs and condensate regulation. This supports emerging models in which changes in the abundance of specific scaffold or client proteins can reshape condensate properties, rather than condensate behavior being governed solely by global changes in translation or RNA availability (Banani et al, 2016; Sankaranarayanan et al, 2021; Bayer et al, 2023). Interestingly, we found that *bruno1* mRNA accumulated in P-bodies after ER stress despite a clear increase in overall Bruno 1 translation. We propose two possible explanations for this observation. First, the marked induction of *bruno1* expression during ER stress may simply result in passive accumulation of a subset of *bruno1* transcripts within P-bodies. Alternatively, once sufficient Bruno 1 protein has been produced to drive changes in condensate organization and promote additional mRNA recruitment, sequestration of excess *bruno1* mRNA into P-bodies may function as a negative feedback mechanism to limit further Bruno 1 protein production. Additional experiments will be required to distinguish between these possibilities.

Functionally, we find that P-body remodeling during ER stress is associated with selective recruitment and stabilization of specific mRNAs, including maternal transcripts and P-body component mRNAs, whereas nonassociated transcripts are degraded. This is consistent with transcriptomic data that shows that upon arsenite stress, the composition of P-bodies drastically changes (Matheny et al, 2019). ER stress activates Ire1-mediated RNA decay through the RIDD pathway, which in *D. melanogaster* has been shown to act broadly on ER-proximal transcripts with limited sequence specificity (Gaddam et al, 2013). In this context, selective sequestration of mRNAs into P-bodies may provide a spatial mechanism to shield essential transcripts from general degradation. Our observation that P-body–associated mRNAs exhibit increased abundance without increased transcription supports a protective, rather than degradative, role for P-bodies under these conditions, consistent with prior observations that P-bodies can function as sites of mRNA storage (Lin et al, 2015; Standart & Weil, 2018).

Finally, our data places P-body remodeling downstream of ATF4, but the mechanism linking ATF4 activity to this process remains to be fully elucidated. Interestingly, P-body remodeling appears to be independent of XBP1, highlighting functional specialization within ER stress signaling pathways. Although XBP1 primarily regulates transcriptional programs that expand ER folding capacity, ATF4 has been implicated in broader adaptive responses, including glycolysis, amino acid metabolism, and stress resistance (Walter & Ron, 2011; Lee et al, 2015; Read & Schröder, 2021). Notably, previous work has found that ATF4 induces increased transcription of 4E-BP, a protein involved in post-transcriptional translation repression (Kang et al, 2017).

Our findings extend the scope of ATF4 signaling to include regulation of RNA:protein condensates and post-transcriptional mRNA fate. Because ATF4 signaling can also be activated by the integrated stress response, these observations raise the possibility that P-body remodeling may participate in stress adaptation beyond ER stress (Pakos-Zebrucka et al, 2016). More broadly, this work suggests that transcriptional stress responses can directly reconfigure the physical organization of the cytoplasm to selectively preserve key gene products during stress, providing a new conceptual link between stress signaling, condensate biology, and mRNA regulation.

## Materials and Methods

### Fly husbandry

*D. melanogaster* stocks were maintained on standard cornmeal agar food at 25°C. Female flies were put in grape vials and fed yeast paste 2–3 d before dissection. Fly stocks obtained from Bloomington *Drosophila* Stock Center were as follows: UAS-*mCherry*^*RNAi*^ (BL #35785)*,* UAS-*bruno1*^*RNAi*^ (BL #35394), *Me31B-GFP* (BL #51530), *tub-a*(V37)-Gal4 (BL #7063), *osk-a* (VP16)-Gal4 (BL #44241), UAS-*XBP1*^*RNAi*^ (BL #36755), UAS-*crc*^*RNAi*^ (ATF4) (BL #80388). Kyoto *Drosophila* Stock Center: *Cup-YFP* (DGRC 115–161). *Rin-GFP* (VDCR #318907) was obtained from the Vienna *Drosophila* Resource Center. *Tral-RFP* was a kind gift from Dr. D. St. Johnston (Gurdon Institute at the University of Cambridge), and UAS-*Bruno 1-GFP* was a kind gift from Dr. M. L. Spletter (University of Missouri-Kansas City). UAS-*mCherry*^*RNAi*^ (BL #35785) was used as a control in all RNAi experiments to account for effects of activated RNAi machinery. All RNAi lines were driven by *tub-a*(V37)-Gal4 (BL #7063) except for UAS-*Bruno-GFP* experiments that required a Gal4 driver on a different chromosome, so *osk-a*(VP16)-Gal4 (BL #44241) was used.

### Immunofluorescence staining of *D. melanogaster* egg chambers

Ovaries were dissected and fixed in 2% PFA in PBS for 10 min at room temperature. Fixed egg chambers were washed three times for 10 min each in PBST (PBS containing 0.3% Triton X-100), then permeabilized and blocked for 2 h in PBS containing 1% Triton X-100 and 1% BSA. Samples were incubated with primary antibodies overnight at room temperature with gentle rocking, followed by three 10-min washes in PBST. Secondary antibody incubation was carried out using fluorescently labeled antibodies (1:1,000; DyLight 550 & 650; Thermo Fisher Scientific) for 2 h at room temperature, followed by an additional three 10-min washes in PBST. Samples were mounted in RapiClear (SunJin Lab) mixed with Aqua-Poly/Mount (Polysciences) at a 75:25 ratio for imaging. The following antibodies and stains were used: mouse anti-Me31B (1:3,000), a generous gift from Dr. A. Nakamura (Institute of Molecular Embryology and Genetics, Kumamoto University), rabbit anti-eIF4G (1:200), a kind gift from Dr. E. Izaurralde (Max Planck Institute for Developmental Biology), and rabbit anti-Bruno 1 (1:10,000), a kind gift from Dr. P. M. Macdonald (University of Texas Austin).

### smFISH labeling

smFISH was performed following the protocol described by Bayer et al (2015), with minor modifications. Ovaries were dissected and fixed in 4% PFA in PBS for 10 min at room temperature. Fixed egg chambers were washed three times for 10 min each in 2x SSC, then prehybridized with a 15-min wash in 2x SSC containing 10% formamide. Samples were then incubated overnight at 37°C with smFISH probes (1:50) and a nuclear membrane stain, wheat germ agglutinin conjugated to CF405S (1:50; Biotium). After hybridization, egg chambers were washed three times for 10 min each in prewarmed 2x SSC with 10% formamide at 37°C and mounted in ProLong Diamond Antifade Mountant (Life Technologies) for imaging. The following probe sets were used: *nanos* mRNA labeled with 48 Quasar 670 probes, *bicoid* mRNA labeled with 48 Quasar 570 probes, *me31B-GFP* mRNA labeled with 30 EGFP recognizing Quasar 670 probes, *cup* mRNA labeled with 48 Cal Fluor Red 590 probes, *armi* mRNA labeled with 48 Cal Fluor Red 590 probes, *glorund* mRNA labeled with 35 Quasar 590 probes, *oskar* mRNA CDS labeled with 48 Quasar 670 probes, *oskar* 5′ mRNA labeled with 46 Quasar 570 probes, *oskar* 3′ mRNA labeled with 45 Cal Fluor Red 610 probes, *bruno1* mRNA labeled with 48 Quasar 670 probes. Probes were made by Biosearch Technologies.

### ER stress induction

Egg chambers were dissected directly into Schneider’s media alone (−DTT) or supplemented with 5 mM DTT (+DTT) or 1 μM thapsigargin (+thapsigargin). After 30-min incubation, egg chambers were rinsed with 1x PBS and then fixed and either mounted as previously described or used for smFISH or immunofluorescence labeling as previously described.

### Microscopy

All imaging was performed using a Leica TCS SP8 Laser Scanning Confocal Microscope equipped with a white light laser (470–670 nm), a 405-nm solid-state laser, and a continuous-wave STED 660-nm high-intensity laser. For confocal imaging, a 63×/1.4 NA oil immersion objective and a 100×/1.4 NA oil immersion objective were used. Optical Z-sections were acquired at 0.3-μm intervals. For STED imaging, a 100x/1.4 NA oil immersion objective was used with a zoom factor of 5x, and optical Z-sections were acquired at 0.22-μm intervals. STED samples were prepared from 25-μm ovary cryosections. All images were acquired using an automated XYZ piezoelectric stage and saved as 16-bit files. Image acquisition was carried out using Leica LAS X software.

### Tissue preparation for sectioning

Ovaries were dissected directly into 4% PFA in 1x PBS and fixed for 10 min at room temperature. Samples were then washed three times for 10 min each in PBST (PBS with 0.1% Triton X-100), followed by a 5-min wash in 0.1 M glycine, pH 3.0. After fixation and washing, ovaries were incubated overnight at 4°C in 30% sucrose. The next day, samples were embedded in O.C.T. Compound (Tissue-Tek) and flash-frozen before sectioning. Cryosections of 25 μm thickness were prepared using a cryotome and stored at −80°C until use.

### Imaging analysis

Identical acquisition settings were used for all control and experimental samples to ensure comparability. For each condition, images were obtained from similar stage egg chambers from three independent experiments, each prepared from a separate fly cross. All raw images were deconvolved before analysis using Leica’s Lightning deconvolution module. Image processing was carried out using consistent batch parameters across all samples. Quantitative analyses were performed using Imaris Microscopy Image Analysis software (Oxford Instruments). Condensates, proteins, and mRNAs were detected using the Imaris “Surface” module, which enables object-based segmentation and object-to-object spatial analysis. Colocalization was determined using the “Surface-Surface” analysis function, where objects with a shortest edge-to-edge distance of less than 0 μm were classified as colocalized. All statistical analyses were conducted using Mann–Whitney statistical tests in GraphPad Prism 8 (GraphPad Software). Figures were assembled and image adjustments applied uniformly using Fiji/ImageJ (NIH) (Schindelin et al, 2012).

### Western blot analysis

For each genotype, 10 ovaries were dissected directly into 95 μl of 2x Laemmli sample buffer (Bio-Rad) supplemented with 5 μl β-mercaptoethanol and immediately subjected to mechanical lysis. Samples were heated at 95°C for 10 min and then centrifuged at 10,000*g* for 10 min at 4°C. Clarified lysates were loaded onto 10% SDS–PAGE for electrophoretic separation. Primary antibodies used in this study included mouse anti-Me31B (1:3,000), generously provided by Dr. A. Nakamura (Institute of Molecular Embryology and Genetics, Kumamoto University); rabbit anti-Tri-methyl-Histone H3 (C42D8) (1:150,000; Cell Signaling Technology); rabbit anti-Bruno (1:10,000), a generous gift from Dr. P. M. MacDonald (University of Texas at Austin); rabbit anti-XBP1 (PB9463) (1:500; Boster Bio). Bands were detected using TrueBlot ULTRA secondary antibodies (anti-mouse and anti-rabbit IgG HRP, 1:50,000; Rockland) and visualized with SuperSignal West Femto Maximum Sensitivity Substrate (Thermo Fisher Scientific).

### RNA isolation and RT–qPCR

Whole ovaries were dissected into ice-cold PBS (4°C) and immediately subjected to mechanical lysis in TRIzol reagent (Thermo Fisher Scientific) for total RNA extraction. RNA was precipitated and washed with ethanol and resuspended in RNase-free water. RNA concentration and purity were assessed before downstream applications. Reverse transcription was performed using 2.5 μg of total RNA with SuperScript IV First-Strand Synthesis Kit (Life Technologies) according to the manufacturer’s instructions. Primers were designed using the DRSC FlyPrimerBank and synthesized by Integrated DNA Technologies. Quantitative PCRs were carried out on a Roche LightCycler 480 system (Roche Molecular Systems, Inc.). Each 10 μl reaction contained 1 μl of cDNA template, 4 μl of 10 μM primer mix, and 5 μl of SYBR Green I Master Mix (Roche Diagnostics). All RT–qPCRs were performed in triplicate. Presented data represent three biological replicates, each consisting of RNA isolated from at least five pairs of ovaries derived from independent fly crosses. Ct values were normalized to Rp49, and resulting normalized expression levels were compared between conditions using two-tailed unpaired Welch’s *t* test.

### Graphics

BioRender was used to prepare Figs 1A and 7G. GraphPad Prism 8 (GraphPad Software) was used to prepare all graphs.

## Supplementary Material

Reviewer comments

## Data Availability

This study includes no data deposited in external repositories.
